# How disability and assistive technologies knowledge might shape transhumanist orientations: a pilot study among Romanian adults

**DOI:** 10.3389/fresc.2026.1786818

**Published:** 2026-06-29

**Authors:** Alexandra Cobzeanu, Bogdan Mihail Cobzeanu, Oana Roxana Bitere-Popa, Iulia Cristina Roca

**Affiliations:** 1 Department of Education Sciences, Faculty of Psychology and Education Sciences, Alexandru Ioan Cuza University, Iași, Romania; 2 Universitatea de Medicina si Farmacie Grigore T Popa Lasi, Iași, Romania

**Keywords:** assistive technologies, disability, disability knowledge, emotions, transhumanism

## Abstract

This pilot study examines disability knowledge, everyday interactions with people with disabilities (PWD), emotional responses to disability, familiarity with assistive technologies, and transhumanist orientation among 863 Romanian adults aged 19 to 45. Results suggested that participants generally reported limited knowledge about disability and assistive technologies, with sadness emerging as the dominant emotional response to disability. Greater disability knowledge was associated with higher knowledge of assistive technologies, which in turn predicted a stronger transhumanist orientation. Assistive technology knowledge mediated the relationship between disability knowledge and transhumanist orientation. Positive interactions with people with disabilities were linked to stronger endorsement of transhumanist orientations. These exploratory findings suggest that assistive technologies may function as a conceptual bridge between social understandings of disability and broader orientations about human enhancement, highlighting the importance of knowledge of assistive technologies in shaping contemporary disability attitudes.

## Introduction

People with disabilities are part of all communities and social worlds. Mainstream disability research increasingly recognizes that disability is shaped not by individual impairment alone, but by the accessibility of environments, social support, institutional arrangements, and cultural meanings (1). From a social and psychosocial perspective, disability is understood as emerging from the interaction between a person and a disabling environment, rather than as an inherent deficit (2). At the same time, disability encompasses a broad range of physical, sensory, cognitive, and mental health conditions that may affect an individual's daily functioning (3).

According to the World Health Organization (4), over one billion people (one in 6 people) globally experience some form of disability, making it a significant social issue that needs our constant attention. In Romania, recent official data indicate that 1 in 20 people holds a disability certificate, i.e., around 900.000 individuals (National Authority for the Protection of the Rights of Persons with Disabilities, 2025). In post-socialist and Eastern European contexts, however, disability has also been shaped by historically specific medical, institutional, and political legacies (5), including segregation (6), uneven deinstitutionalization, and persistent tensions among care, control, and inclusion.

It is a well-known fact that people with disabilities (PWD) often experience stigma, discrimination, devaluation, and generally negative attitudes (7). At the same time, research highlights that the frequency and quality of interactions with PWD are some of the primary factors that influence our attitudes toward disability (8). Thus, examining and understanding the role of disability interactions is highly important for fostering inclusion and reducing stigma, since these interactions can shape societal attitudes (9, 10), influencing policies and technological advancements aimed at improving their quality of life (11). Furthermore, examining these interactions can provide important insights into public perceptions, emotional responses (12), and the role of assistive technologies in bridging accessibility gaps (13). The present study examines how self-assessed disability-related knowledge, everyday interactions with people with disabilities, emotional responses to disability, and knowledge of assistive technologies are associated with transhumanist orientation (i.e., the belief that science and technology can and should enhance human abilities beyond current biological limits (14); in a Romanian adult sample.

## The role of emotions in disability perception

Emotions are highly important in shaping how people perceive and respond to disabilities (52), as they constitute a specific component of attitudes in this regard (15). Emotional responses play a key role in shaping the relationship between contact and attitudes (16), as well as the desire to maintain social distance from an out-group (17). Higher contact is associated with reduced inter-group anxiety and more favorable attitudes toward the out-group (18). At the same time, greater familiarity with an out-group is associated with fewer negative emotional reactions and a lower tendency to seek social distance (17).

Emotional responses to disability vary widely, ranging from fear and sadness to happiness and admiration (19). For instance, Hughes (20) emphasized that PWD have historically been associated with disgust, pity, and fear. Consequently, they often become the target of conflicting emotions from the broader non-disabled society, including hostility and negative attitudes in general (21). Conversely, positive emotions associated with disability might lead to more positive attitudes and reduced stigma.

Furthermore, emotional responses toward PWD can be better understood when grouped into broader functional categories rather than treated as isolated reactions. For instance, threat-based emotions, such as fear, discomfort, or anxiety, are typically linked to avoidance and social distancing (22), whereas affiliative emotions, including empathy and compassion, are associated with prosocial intentions (23) and, implicitly, support for inclusion (24). Sadness, however, is somehow ambiguous, as it may reflect concern and emotional engagement but can also reinforce paternalistic or charitable views of disability (25). When sadness becomes the dominant response, it may risk framing people with disabilities primarily as objects of suffering rather than as autonomous individuals (26). Thus, examining the emotions associated with disability is important both for understanding prejudice and also for identifying responses that may appear positive while still limiting genuine inclusion.

## Knowledge of assistive technologies and transhumanist orientation

Assistive technologies play a transformative role in enhancing the lives of PWD (27). These technologies might include mobility aids (e.g., wheelchairs, prosthetic limbs), communication devices (e.g., speech-to-text software), and sensory enhancements (e.g., cochlear implants for hearing impairments) (28). By integrating cutting-edge advancements in artificial intelligence, robotics, and biomedical engineering, assistive technologies can help PWD achieve greater independence and improve their quality of life (29, 30). However, assistive technologies for PWD are not consistently available in many areas due to economic, educational, or social factors (31). In Romania, consistent efforts are being made in this regard, but progress still takes considerable time and resources (32). In the present study, knowledge of assistive technologies is not treated as equivalent to endorsement of transhumanism as a comprehensive ideology, but rather as a concrete, applied domain through which broader orientations toward technological intervention and human enhancement may be shaped.

Transhumanist orientation refers to the belief that advanced technology and science can and should be used to enhance human abilities and overcome biological limitations. As Szabados (33) notes, transhumanism can be considered “a product of the ongoing scientific and technological revolution” (p. 1). People with a stronger transhumanist orientation have a more positive view of technological interventions, such as biomedical enhancements and assistive and augmentative technologies, considering them as desirable tools for improving physical, cognitive, or emotional functioning for people with various illnesses, disabilities, and even in the case of aging (34). However, because transhumanism seeks to alter fundamental evolutionary processes through practices such as cloning, gene therapies, mind uploading, or nanotechnology, it remains a highly controversial perspective, with higher support among men and younger individuals (14).

There is an emerging body of literature documenting transhumanism in relation to technological progress and human enhancement (35). However, its role in understanding perceptions of disability and attitudes toward PWD, especially in a transitioning country such as Romania, has not received as much empirical attention. Examining transhumanist orientation is important in this context because it reflects orientations about whether human limitations, including, for example, the physical limitations some disabilities entail, should be accommodated or technologically altered, thereby placing it at the intersection of medical and social models of disability (36). These orientations are likely to shape how people might evaluate and report on assistive technologies, either as tools for inclusion and support or as means of “correcting” bodily difference (37). While existing research has shown that limited or negative contact and low disability-related knowledge contribute to negative attitudes toward PWD (8, 38), these factors have not yet been examined in relation to disability knowledge, attitudes toward assistive technologies, and transhumanist orientation, as our study aims to do.

Although a transhumanist orientation emphasizes technological progress and the expansion of human capabilities, it is normatively at odds with disability studies perspectives (39). Some voices say that transhumanist narratives may endorse ableism by portraying disability primarily as a limitation to be overcome or eliminated through technological intervention/enhancement. From this viewpoint, an emphasis on “fixing” or enhancing bodies risks devaluing disabled identities and overlooking the social and structural barriers that produce disability in the first place (40). In other words, transhumanist orientations may support assistive technologies that enhance autonomy but may also reinforce medicalized understandings of disability (41).

### The present study

The present study addresses an important gap in the literature, particularly in the Romanian context, where empirical research on disability attitudes toward transhumanism and human enhancement remains limited. In Romania, public perceptions of disability are often shaped by restricted social exposure, persistent stigma, and uneven access to inclusive education and assistive technologies (6, 42). Against this background, our study aims to examine how individuals' interactions with people with disabilities might be linked to their emotional responses and disability-related knowledge, how such knowledge relates to attitudes toward assistive technologies in a society with developing support infrastructures, and whether endorsement of transhumanist ideas might be associated with particular perceptions of disability among Romanian participants.

We aimed to test the following hypotheses: ***H1*:** Greater knowledge of disabilities would be associated with higher knowledge of assistive technologies. Our assumption is primarily based on the guidelines provided by intergroup contact theories, which highlights that increased knowledge and familiarity with “outgroup” members (in our case, PWD) may reduce and negative affect while fostering more nuanced emotional responses, higher empathy and acceptance (43), as well as more information regarding the group—in this case, information about assistive technologies [we use “out-group” as a technical term borrowed from intergroup contact theory, while acknowledging that framing PWD as an out-group risks overgeneralization]; ***H2***: Higher knowledge about assistive technologies would be associated with a stronger transhumanist orientation, particularly when assistive technologies are framed as tools for autonomy, dignity, and participation rather than as markers of deficit (44); ***H3***. Positive interactions with people with disabilities would be positively associated with transhumanist orientation. This third assumption is based on theories of social experience and belief formation, which suggest that repeated interpersonal encounters shape broader ideological attitudes (45). Thus, positive interactions may normalize bodily and cognitive diversity, fostering more inclusive and socially oriented interpretations of technology use. Finally, we assumed that ***H4****.* Age would be negatively related to transhumanist orientation. More specifically, we assumed that younger individuals would be more likely to embrace transhumanism, as previous studies suggest that relatively older individuals, even within adult samples, are generally more reluctant to adopt new technologies (46, 47), especially those with a controversial background, such as these.

## Method

### Participants and procedure

Romanian adults aged 19 to 45 years were recruited. The target was to obtain a diverse sample of adults through snowball sampling on social media and professional networks. All participants gave informed consent online, prior to beginning the survey, confirming that their participation was voluntary. All participants were Romanian adults residing in Romania. The sample was restricted to individuals without disabilities. The study was conducted in accordance with institutional ethical standards, as approved by the Ethics Review Committee of the Faculty of Psychology and Education Sciences, Alexandru Ioan Cuza University of Iași (UAIC Iași; ethical approval no. 154/16.01.2025). Participants were recruited via a snowball sampling approach on social media platforms and professional networks to achieve a diverse sample. The survey was conducted online in Romanian using Google Forms. Data collection took place at the beginning of 2025. The time needed to answer all the questions was about 10 min.

### Measures

After answering questions about their age, gender, and educational level, the participants were asked about their interactions with people with disabilities, their emotional responses to disability, their knowledge of assistive technologies, and their transhumanist orientations. More specifically, participants answered the following questions: (Q1) *Which is the disability you interact with most daily*? (open-ended question, not mandatory); (Q2) *Which is the disability you have interacted with the most at work*? (open-ended question, not mandatory); (Q3) *How would you rate your level of **disability knowledge***? (1 = very low, 5 = very high); (Q4) *How would you rate the **valence*** [i.e., the overall positive or negative quality of the interaction] ***of the interaction*** *with people with disability, in general?* (1 = very negative, 5 = highly positive); (5) *What **emotion** do you mostly feel **when you think** about disability*? (fear/happiness/sadness/surprise/disgust/anger); (6) *What is the **emotion** you mostly feel **when interacting** with a person with a disability*? (fear/happiness/sadness/surprise/disgust/anger); (7) *How would you rate your **knowledge regarding assistive technologies** for people with disabilities*? (1 = very low, 5 = very high). These questions were developed by the research team, drawing on standard formulations commonly used in Romanian survey-based research on disability. Emotional responses to disability (Q5 and Q6) were assessed using six basic emotion categories derived from Ekman's (48) framework, which was considered appropriate for this exploratory pilot study. Questions 1 and 2 were open-ended and completed in Romanian; responses were subsequently translated into English for analysis and categorized by the research team. The category labels (e.g., “locomotory”) reflect standard formulations commonly used in Romanian survey-based research on disability.

#### Transhumanist orientation

Next, they filled out the 5-item Transhumanist Orientation Scale proposed by Brooks et al. (14). The five items (e.g., “I believe people should use nanotechnology to enhance human abilities”) were rated on a 5-point Likert scale from 1 (totally disagree) to 5 (totally agree). Higher scores indicated a higher transhumanist orientation. Cronbach's alpha for this study was 0.85.

Age was treated as a continuous variable reflecting relative age differences within an exclusively adult sample.

### Statistical analyses

Statistical analyses were conducted using standard statistical software (SPSS, v.26) and the PROCESS macro (Model 4). Descriptive statistics were calculated for all variables. Pearson correlation coefficients were used to examine bivariate associations. A mediation analysis with 5,000 bootstrap samples and 95% confidence intervals was conducted to test whether knowledge of assistive technologies mediated the relationship between disability knowledge and transhumanist orientation, controlling for age.

## Results

The final research sample comprised 863 Romanian adults aged 19 to 45 (*M*age = 23.36, *SD* = 5.89). Most participants were female (62.7%) and held a high school diploma (62.2%) or a Bachelor's degree (27.7%).

Regarding the first question, i.e., *Which is the disability you most interact with daily*?, most participants answered that locomotory disabilities are the most encountered, followed by autism spectrum disorders (ASD) and Down Syndrome. For the second question, i.e., *Which is the disability you most interacted with at work*?, the answers were quite similar: most participants answered that locomotory disabilities are the most encountered, followed by ASD and visual impairment. Most participants rated their knowledge of disability as medium (39.2%) or weak (23.4%), and their experiences with people with disabilities as relatively neutral in valence (i.e., whether the interaction was experienced as positive or negative; 34.3%). In terms of emotions, most participants reported feeling sadness when thinking and interacting with disability (see Tables 1–3). Finally, regarding their knowledge of assistive technologies for people with disabilities, most participants reported having weak or moderate knowledge (29.2%).

**Table 1:** Q1. Which is the disability you most interacted with daily? (*n* = 616 answers).

Type	*n*	%
Locomotory	222	36.03
Autism	189	30.68
Down Syndrome	51	8.28
ADHD	37	6.01
Intellectual	34	5.52
Visual	32	5.20
Mental illness	15	2.44
Hearing	13	2.11
Learning	7	1.14
Amputations	7	1.14
Diabetes	4	0.65
Dementia	4	0.65
Speech	1	0.15

Percentages are based on the total number of responses for each question (*n* = 616 for Q1; *n* = 661 for Q2). Participants who did not answer are not included (*n* = 247 for Q1; *n* = 202 for Q2). Each participant selected one response. Category labels reflect participants’ own responses to an open-ended question, translated from Romanian. The survey used the term “locomotory”; the equivalent term “mobility impairments” is used in the main text for consistency with the broader literature.

**Table 2:** Q2. Which is the disability you most interacted with at work? (*n* = 661 answers).

Type	*n*	%
Locomotory	264	39.96
Autism	169	25.58
Visual	43	6.50
Intellectual	41	6.20
Down Syndrome	34	5.14
ADHD	33	4.99
Mental illness	28	4.23
Hearing	13	1.97
Learning	10	1.51
Speech	9	1.36
Anxiety	5	0.76
Sclerosis	3	0.45
Amputations	3	0.45
Epilepsy	2	0.30
Dementia	2	0.30
Diabetes	1	0.15
Depression	1	0.15

Percentages are based on the total number of responses for each question (*n* = 616 for Q1; *n* = 661 for Q2). Participants who did not answer are not included (*n* = 247 for Q1; *n* = 202 for Q2). Each participant selected one response. Category labels reflect participants’ own responses to an open-ended question, translated from Romanian. The survey used the term “locomotory”; the equivalent term “mobility impairments” is used in the main text for consistency with the broader literature.

**Table 3:** (*n* = 863 answers).

Variable	Fear	Happiness	Sadness	Disgust	Surprise	Anger
	%					
Q5. What emotion do you mostly feel when you think about disability?	8.8	6.3	74.9	0.7	8.7	0.7
Q6. What is the emotion you mostly feel when interacting with a person with a disability?	4.4	28.7	51.1	0.8	14.8	0.1

The correlation analysis indicated a strong positive correlation between knowledge of disabilities and knowledge of assistive technologies (*r* = .60, *p* < .001), confirming the first assumption (H1). Our data also indicated a positive correlation between knowledge of disabilities and transhumanist orientation, *r* = .07, *p* = .03, confirming the second assumption (H2). Further, the valence of interactions with people with disabilities was positively correlated with transhumanist orientation (*r* = .12, *p* < .001): the more positive the interactions, the higher the transhumanist orientation. Finally, results indicated a negative but non-significant relation between age and transhumanist orientation.

We further conducted, at an exploratory level—given the pilot nature of this study, a mediation analysis using PROCESS Model 4 (with 5,000 bootstrap samples, 95% bootstrap confidence interval) to examine whether knowledge about assistive technologies mediates the relationship between knowledge about disability and transhumanist orientation, while controlling for age. Results suggested that knowledge about disability significantly and positively predicted knowledge about assistive technologies (*b* = 0.62, SE = 0.03, *t* = 21.98, *p* < .001, 95% CI [0.56, 0.67]). The model predicting transhumanist orientation from knowledge about disability, knowledge about assistive technologies, and age was also significant, *R*² = .018, F(3, 859) = 5.11, *p* = .002. Further, participants' knowledge about assistive technologies significantly predicted transhumanist orientation, *b* = 0.85, *SE* = 0.29, *t* = 2.95, *p* = .003, 95% CI [0.28, 1.42]. The direct effect was not significant when the mediator was included, *b* = 0.01, *SE* = 0.30, *t* = 0.05, *p* = .96, but the indirect effect was statistically significant, *b* = 0.53, *SE* = 0.19, 95% CI [0.17, 0.91]. Thus, our data indicate full mediation: greater knowledge about disability is associated with a higher transhumanist orientation, indirectly through increased knowledge of assistive technologies.

## Discussion

In the present study, we conducted an exploratory, pilot-level examination of how Romanian adults' knowledge about disability, familiarity with assistive technologies, and transhumanist orientation might be related. We also examined which disabilities are most commonly encountered in participants' everyday lives, which primary emotions are experienced when thinking about and interacting with PWD, and the valence of those interactions. Participants reported that their most frequent daily and professional interactions involved locomotory disabilities (i.e., mobility-related conditions), followed by ASD and Down syndrome in everyday contexts, and by ASD and visual impairment in work-related settings. These patterns suggest that participants' representations of disability are shaped primarily by more visible or commonly encountered conditions, which may influence both emotional responses and knowledge structures. These results also align with previous studies conducted within the Romanian population. For instance, the research of Maftei and Gherguț (49) highlighted that, similar to the present study, locomotory disabilities were the most commonly reported disability category among adults with disabilities, whereas ASD was most frequently associated with children with disabilities.

Further, self-assessed knowledge about disability was relatively modest. More specifically, most participants rated their knowledge as medium or weak, and their experiences with people with disabilities were generally described as emotionally neutral. Nevertheless, sadness was the dominant emotion associated with thinking about and interacting with disability. These two specific results may suggest that disability is approached more through affective and symbolic frameworks than through informed understanding, a dynamic that may limit how people understand technological interventions/enhancements in the context of disability.

In the proposed exploratory mediation model, greater knowledge about disability was associated with higher knowledge of assistive technologies, and this technological knowledge, in turn, was linked to a stronger transhumanist orientation. This finding suggested that the relation between disability knowledge and transhumanist orientation may be fully mediated by knowledge of assistive technologies. In other words, familiarity with disability alone doesn't seem sufficient to predict transhumanist views unless it is accompanied by an understanding of concrete technological solutions. We believe this result may be highly important for future research, as it suggests that assistive technologies may serve as a conceptual bridge between social experiences of disability and broader orientations about human enhancement.

From a theoretical perspective, the present findings suggest that adults' attitudes toward emerging technologies may be less influenced by abstract ideas and more shaped by applied, context-specific knowledge. At the same time, the emotional pattern observed in the sample, i.e., the dominance of sadness in responses to disability, may suggest that disability is still largely framed through a lens of vulnerability, and this may partly explain why assistive technologies emerge as a strong mediator: they may represent hope, agency, or progress considered otherwise absent in prevailing representations of disability. However, this is a speculative explanation that needs further exploration in future studies.

Also, critical perspectives from disability studies caution that transhumanist narratives may reinforce ableist or medicalized views of disability by framing bodily difference primarily as a limitation to be technologically overcome, potentially devaluing disability identities in favor of normalization and enhancement (39–41). However, in the present study, the observed association between assistive technology knowledge and transhumanist orientation is not interpreted as endorsement of disability erasure, but rather as reflecting how concrete technological knowledge may shape broader orientations about human enhancement. Given the exploratory and quantitative nature of this research, identity-based critiques of transhumanism could not be directly examined and remain an important direction for future qualitative work.

On a more practical note, our findings highlight the need for more specific, multidimensional measures of disability knowledge and knowledge of assistive technologies. Second, longitudinal or experimental designs might help us better understand how knowledge about assistive technologies shapes transhumanist attitudes, or whether individuals with pre-existing pro-technology orientations seek out such knowledge. Third, future work should engage more directly with disability studies perspectives, examining whether transhumanist orientations promote inclusion or risk reinforcing medicalized and ableist narratives (41, 50). At the same time, expanding this line of research across different cultural contexts would also help us understand whether the patterns we observed are context-specific or more universal.

Several limitations need to be accounted for when interpreting these findings. The most important point is that this research was explicitly designed as a pilot and exploratory investigation, relying on self-reported, single-item measures for several constructs and a cross-sectional design that does not allow us to draw causal conclusions. The Romanian sociocultural context is characterized by uneven access to assistive technologies and broader technology inequality (51), along with limited public discourse on transhumanism, which constrains the generalizability of the present results. Furthermore, another limitation concerns the assessment of emotional reactions to disability. Emotions were evaluated using a limited selection of fundamental emotions based on Ekman's framework (48), an approach we considered appropriate for an exploratory pilot study, although it constrained the evaluation of affiliative or respect-oriented reactions such as empathy, compassion, or acceptance. The prevalence of sadness should therefore be approached with caution, since it may indicate concern-driven or paternalistic perspectives rather than explicit emotions. Finally, because several constructs were measured using brief self-report items, future research should more explicitly distinguish among biomedical knowledge, social knowledge, rights-based knowledge, and experiential familiarity with disability.

## Conclusion

Although limited by its exploratory nature, our pilot study highlights the potentially important role of knowledge-assistive technologies in shaping how people understand disability and imagine technologically shaped futures.

## Data Availability

The raw data supporting the conclusions of this article will be made available by the authors, without undue reservation.
